# A rare form of urinary tract infection in a transplanted patient

**DOI:** 10.1590/2175-8239-JBN-2024-0125en

**Published:** 2024-12-20

**Authors:** Nídia Marques, Adriana Santos, Filipa Ferreira

**Affiliations:** 1 Unidade Local de Saúde São João, Departamento de Nefrologia, Porto, Portugal. Unidade Local de Saúde São João Departamento de Nefrologia Porto Portugal

**Figure 1.**
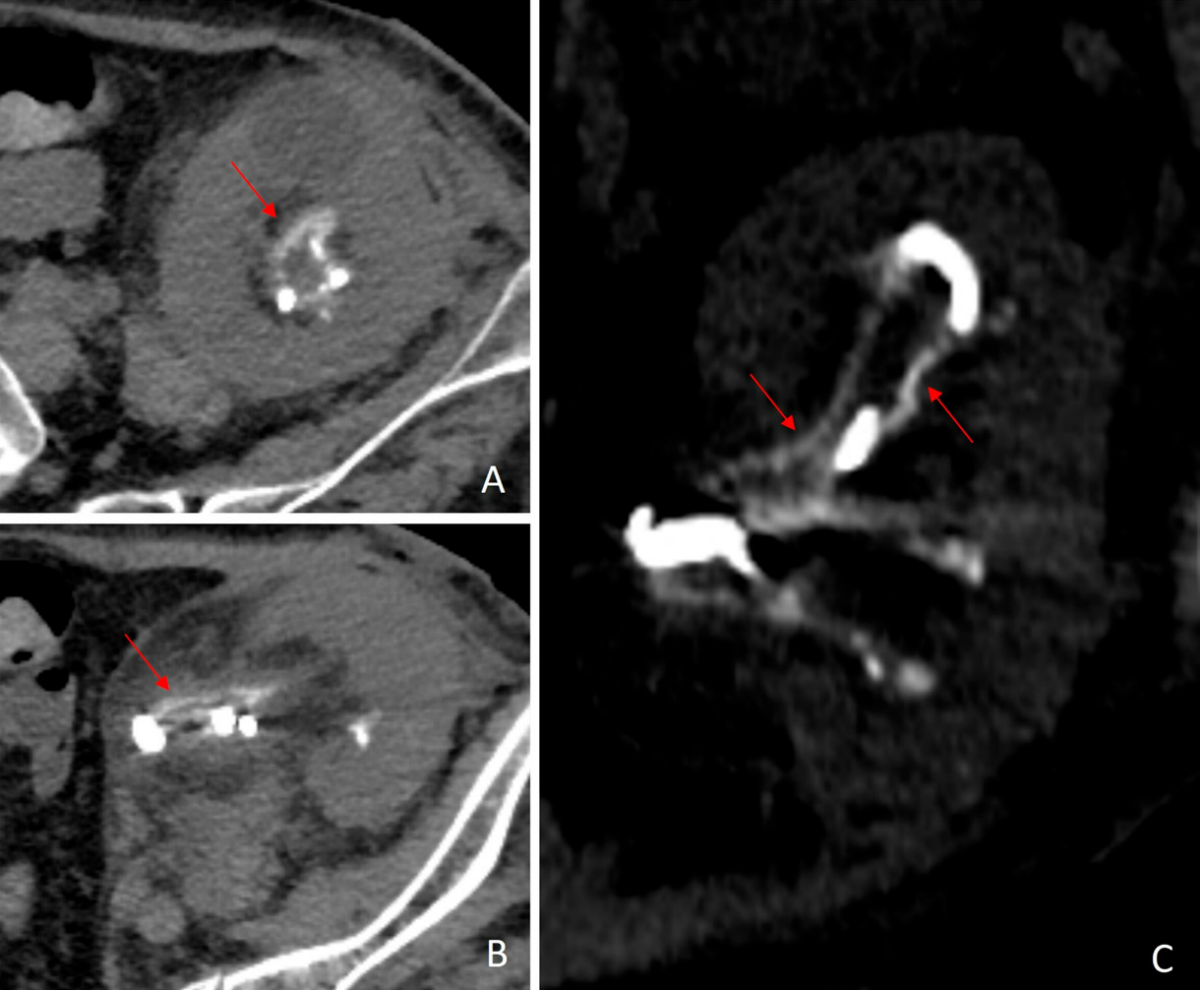
CT shows thick linear urothelial calcification (arrow), suggestice of alkaline-encrusted pyelitis. This condition is associated with an immunosuppressed state, previous urologic procedures, and pre-existing urothelial lesions.

A 62-year-old woman with a second kidney transplant underwent surgery to reimplant the ureter after a fistula of the ureter-bladder anastomosis was detected. A peri-allograft collection was drained during the procedure, and a *Corynebacterium urealyticum* was identified in culture. A computed tomography scan revealed thick linear urothelial calcification suggestive of alkaline-encrusted pyelitis (Figure 1). The patient was initiated on intravenous linezolid and high doses of ascorbic acid and Suby G (citric acid monohydrate, mild magnesium oxide, sodium bicarbonate, edetate disodium, and water, pH 7.4) via percutaneous nephrostomy. Urea-splitting agents cause alkaline-encrusted pyelitis, a rare urinary tract infection that can be difficult to diagnose due to its fastidious growth in common cultural conditions.^1,2^
