# Supplementary material for: A rare form of urinary tract infection in a transplanted patient
Source: J Bras Nefrol. 2024 Dec 20;47(2):e20240125. doi: 10.1590/2175-8239-JBN-2024-0125en (PMC11660445; doi:10.1590/2175-8239-JBN-2024-0125en)
Supplement: Translation

# Translation: Uma forma rara de infecção do trato urinário em paciente transplantado

**DOI:** 10.1590/2175-8239-JBN-2024-0125pt

**Authors:** Nídia Marques, Adriana Santos, Filipa Ferreira

**Affiliations:** 1 Unidade Local de Saúde São João, Departamento de Nefrologia, Porto, Portugal. Unidade Local de Saúde São João Departamento de Nefrologia Porto Portugal

Uma mulher de 62 anos, com segundo transplante renal, foi submetida a cirurgia para reimplante do ureter após detecção de uma fístula na anastomose ureterovesical. Durante o procedimento, foi drenada uma coleção de peri-aloenxerto e identificou-se *Corynebacterium urealyticum* na cultura. Uma tomografia computadorizada revelou calcificação urotelial linear espessa, sugestiva de pielite incrustada alcalina (Figura 1). A paciente iniciou tratamento com linezolida intravenosa e altas doses de ácido ascórbico e Suby G (ácido cítrico monohidratado, óxido de magnésio leve, bicarbonato de sódio, edetato dissódico e água; pH 7,4) via nefrostomia percutânea. Agentes decompositores de ureia causam pielite incrustada alcalina, uma infecção rara do trato urinário que pode ser de difícil diagnóstico devido ao seu crescimento fastidioso em condições culturais comuns.^1,2^

**Figura 1.** A TC mostra calcificação urotelial linear espessa (seta), sugestiva de pielite incrustada alcalina. Essa condição está associada a um estado de imunossupressão, procedimentos urológicos anteriores e lesões uroteliais preexistentes.
